# Hand Hygiene Behaviors in a Representative Sample of Polish Adolescents in Regions Stratified by COVID-19 Morbidity and by Confounding Variables (PLACE-19 Study): Is There Any Association?

**DOI:** 10.3390/pathogens9121011

**Published:** 2020-12-01

**Authors:** Dominika Skolmowska, Dominika Głąbska, Dominika Guzek

**Affiliations:** 1 Department of Dietetics, Institute of Human Nutrition Sciences, Warsaw University of Life Sciences (SGGW-WULS), 159C Nowoursynowska Street, 02-776 Warsaw, Poland; dominika_skolmowska@sggw.edu.pl; 2 Department of Food Market and Consumer Research, Institute of Human Nutrition Sciences, Warsaw University of Life Sciences (SGGW-WULS), 159C Nowoursynowska Street, 02-776 Warsaw, Poland; dominika_guzek@sggw.edu.pl

**Keywords:** hand hygiene, hand washing, adolescents, regions, Polish representative sample, COVID-19 morbidity, Coronavirus-19, COVID-19, SARS-CoV-2, Polish Adolescents’ COVID-19 Experience (PLACE-19) Study

## Abstract

The hand hygiene may possibly influence the course of the COVID-19 pandemic, but the multifactorial influence on hand hygiene knowledge and behaviors is proven. The aim of the study was to analyze hand hygiene behaviors in a national representative sample of Polish adolescents in regions stratified by COVID-19 morbidity, while taking socioeconomic status of the region, as well rural or urban environment, into account as possible interfering factors. The study was conducted Polish Adolescents’ COVID-19 Experience (PLACE-19) Study population (*n* = 2323) that was recruited based on a random sampling of schools, while the pair-matching procedure was applied within schools and age, in order to obtain adequate number of boys and girls, representative for the general Polish population (*n* = 1222). The participants were asked about their handwashing habits while using Handwashing Habits Questionnaire (HHQ) and about applied procedure of washing hands. The results were compared in subgroups that were stratified by region for COVID-19 morbidity, socioeconomic status of the region, and rural/urban environment. In regions of low COVID-19 morbidity, a higher share of adolescents, than in regions of high morbidity, declared washing their hands before meals (*p* = 0.0196), after meals (*p* = 0.0041), after preparing meals (*p* = 0.0297), before using the restroom (*p* = 0.0068), after using the restroom (*p* = 0.0014), after combing their hair (*p* = 0.0298), after handshaking (*p* = 0.0373), after touching animals (*p* = 0.0007), after contacting babies (*p* = 0.0278), after blowing nose (*p* = 0.0435), after touching sick people (*p* = 0.0351), and after cleaning home (*p* = 0.0234). For the assessed steps of the handwashing procedure, in regions of low COVID-19 morbidity, a higher share of adolescents included them to their daily handwashing, than in regions of high morbidity, that was stated for removing watch and bracelets (*p* = 0.0052), removing rings (*p* = 0.0318), and drying hands with towel (*p* = 0.0031). For the comparison in regions stratified by Gross Domestic Product, the differences were only minor and inconsistent. For the comparison in place of residence stratified by number of residents in city, there were some minor differences indicating better hand hygiene behaviors in the case of villages and small towns when compared with medium and large cities (*p* < 0.05). It may be concluded that, in a population-based sample of Polish adolescents, individuals from regions of low COVID-19 morbidity presented more beneficial hand hygiene habits than those from regions of high COVID-19 morbidity.

## 1. Introduction

In December 2019, a novel SARS-CoV-2 coronavirus, which caused the Coronavirus Disease 2019 (COVID-19) [1], occurred in Hubei Province, China [2]. The virus soon affected other countries, so, taking into consideration a significant increase in number of cases, the virus has been declared by World Health Organization (WHO) a public health emergency of international concern [3] and a pandemic [4]. A range of various preventive measures were implemented, such as lockdown, home isolation, quarantine, and social distancing, because it was stated that the virus is transmitted from human-to-human by droplets through coughing and sneezing [5,6,7]. The effectiveness of such approach was confirmed by a meta-analysis by Chu et al. [8], meta-analysis by Liang et al. [9], and other analysis [10,11,12]. Moreover, the importance of applying proper hand hygiene behaviors was highlighted, as human’s hands pose a critical vector for the transmission of pathogens [13,14], so a proper hygiene [15], including frequent hand washing with water and soap, or using hand sanitizer with at least 60% alcohol were recommended [7].

It is pointed out that high-risk groups, which are especially susceptible to the virus infection, are mainly elderly people and immunocompromised ones [16], while children and adolescents are less commonly diagnosed as infected [17] and they tend to have mild COVID-19 course when comparing to adults [18]. However, some authors indicated that within groups especially vulnerable to the virus infection are also adolescents of poor hand hygiene behaviors [19]. At the same time, adolescents may play an important role in the virus spreading, as, in the Korean study conducted by Park et al. [20], it was shown that they were most likely group to spread the virus in their households.

Despite recommending frequent hand washing by all major authorities that are responsible for international public health, such as WHO [21] or Centers for Disease Control and Prevention (CDC) [22], among children and adolescents there is still an inadequate level of knowledge concerning proper hand washing procedures. In the study of Chen et al. [23] that was conducted in a group of primary school students from Wuhan, China during the COVID-19 pandemic, it was revealed that only 42% of them displayed correct hand-washing behaviors.

It is known that the adherence to the hygiene behaviors is influenced by a number of factors, including age [24,25,26], gender [27,28,29], socioeconomic status [30,31,32], and educational level [25]. It is commonly indicated that age may be associated with hand hygiene, but the results depend on the population group, as, depending on the study, a young [25], middle aged respondents [26], or older ones [24] are characterized by the best hand hygiene knowledge and behaviors. At the same time, a majority of studies indicate better hand hygiene in females than in male respondents [27,28,29]. Moreover, poor hand washing practices are indicated as being associated with lower socioeconomic status [30,31,32] and lower level of education [25]. Among the other determinants influencing hand washing behaviors, there may be a place of residence, as in some studies, it was found that respondents living in the urban areas were characterized by significantly better hand washing behaviors than those living in the rural areas [33,34].

For the COVID-19 issue, there may also be an association with hand hygiene, as the meta-analysis by Aiello et al. [35] indicated that an improvement in hand hygiene results in a reduction in respiratory illness of 21%. However, the phase of pandemic may also influence the association, as, in the study by Moore et al. [36], conducted in hospitals, hand hygiene first increased as the pandemic began and then decreased as it progressed.

Taking into account the multifactorial influence on hand hygiene and possible influence on the course of COVID-19 pandemic, it may be hypothesized that hand hygiene behaviors are associated with the place of residence and the COVID-19 morbidity in the region, while the other characteristics of the place of residence, including socioeconomic status of the region, and rural or urban environment, may interfere. An understanding of the hand washing adherence, depending on the place of residence, may enable to create effective educational campaign addressed to the certain population group, including especially children and adolescents. Considering it, the aim of the presented study was to analyze hand hygiene behaviors in a national representative sample of Polish adolescents in regions that are stratified by COVID-19 morbidity, socioeconomic status of the region, and rural or urban environment within the Polish Adolescents’ COVID-19 Experience (PLACE-19) Study population.

## 2. Materials and Methods

### 2.1. Study Population

The study group was randomly chosen within the first phase of PLACE-19 study in a national sample of adolescents from all regions of Poland. The first phase focused on the hand hygiene and personal protective behaviors (conducted in April 2020) [37,38], while the second phase focused on eating behaviors and dietary habits (conducted in May 2020) [39]. For the first phase, the respondents were recruited while using a two-stage stratified sampling procedure and random quota sampling with quotas for regions of Poland was applied:

- first stage (conducted for all 16 voivodeships, which are basic administrative units in Poland and are further divided into counties, in the period of 31 March–14 April 2020)—random selection of five counties out of each of the voivodeship and five secondary schools out of each of the county, resulting in random selection of 400 secondary schools from Poland;
- second stage (conducted for 10 voivodeships, in which after the first stage a total number of obtained answers was insufficient, in the period of 15 April–29 April 2020)—random selection of five counties out of each of the voivodeship and five secondary schools out of each of the county, resulting in random selection of 250 secondary schools from Poland.

The total number of the COVID-19 cases in Poland, by the end of April 2020, was 12,640, based on the statistics by WHO [40]. Table 1 presents the weekly data for the cumulative number of the COVID-19 cases in the voivodeships in Poland in April 2020 [41], based on the Polish statistics.

The headmaster of each randomly chosen school received an invitation to participate in the study, as well as information regarding the aim and the scope of the study. If she/he agreed to participate in the study, the students, who volunteered to participate, must have provided written informed consent from themselves and their parents to take part in the study. As soon as informed consents were gathered, the headmaster forwarded a link to the online questionnaire that was dedicated for the students. Students filled out the questionnaire in any time and place convenient for them up to two weeks after receiving a link. Because some of the headmasters did not give information regarding whether they are willing to participate in the study, a reminder was sent to them after one week of the first, as well as of the second stage of the study.

The inclusion criteria were, as follows: adolescents aged 15–20, attending randomly selected secondary schools. The exclusion criteria included any unreliable answers or missing data.

On the basis of the described procedure, 2323 secondary school students were recruited and participated in the study. The obtained sample was stated to be population-based because the procedure involved the recruitment of the adolescents from all the regions of Poland, while the Net Enrollment Rate (NER) for this age group is calculated as 89.38%, according to the data of Polish Central Statistical Office (CSO) [42].

**Figure 1:**
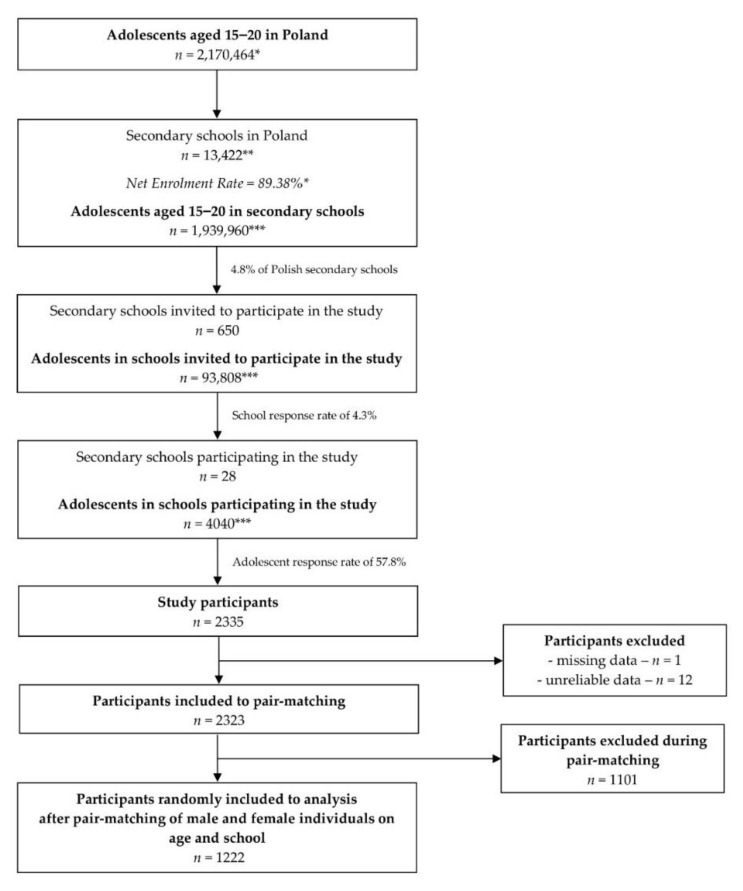
The procedure of sampling, recruitment and inclusion for the national-based sample of Polish adolescents within PLACE-19 study; * data based on Central Statistical Office (CSO); ** calculation based on CSO data; *** calculated based on Central Statistical Office (CSO) data.

After recruiting individuals, they were included to the analysis based on a randomized pair-matching procedure that was applied within schools and age, to obtain adequate number of boys and girls, representative for the general Polish population (*n* = 1222). Figure 1 presents the detailed procedure of sampling, recruitment, and inclusion.

### 2.2. Applied Questionnaire

The study was carried out in the period of remote learning, which was introduced due to the decision of Polish Ministry of National Education [43]. The questionnaire that was used consisted of two sections. First section included questions regarding the general characteristics of respondents, which enabled the verification of inclusion criteria. The respondents were asked about their sex (closed-end question), age (open-ended question), and attended school (open-ended question), as only adolescents aged 15–20 and attending randomly chosen school were allowed to participate in the study. Second part concerned questions regarding hand hygiene behaviors. An electronic questionnaire was used to obtain the data. There was no possibility to identify none of the respondents, as all data that were gathered were anonymous.

The respondents were asked about their current hand hygiene behaviors, being typical for the period of the COVID-19 pandemic. The questions focused on the circumstances of washing hands and the handwashing procedure. The circumstances of washing hands were evaluated on the basis of the commonly used Handwashing Habits Questionnaire (HHQ) [44,45,46], which was developed by Tüzün et al. [47], on the basis of the previous questionnaire developed by Üner et al. [48]. The circumstances of washing hands were distinguished and, for each of them, the respondents were asked to clarify whether they wash their hands in specific situations, with categories, as follows: always, sometimes, never. For some situations there was an additional answer indicating that this step is not applicable for the respondents. The circumstances of washing hands were listed, as follows: before and after meals, before and after preparing meals (both with an additional answer “not applicable”), before and after using the restroom, before going to bed and after waking up in the morning, after combing their hair (with an additional answer “not applicable”), after coming back home, after using public transportation, after money exchange, when hands are visibly soiled, after handshaking, after touching animals (with an additional answer “not applicable”), after handling animal waste and animal food (both with an additional answer “not applicable”), after contacting babies and changing diapers (both with an additional answer “not applicable”), after blowing nose, after sneezing, after coughing, before and after touching sick people, after touching garbage (with an additional answer “not applicable”), after cleaning their home (with an additional answer “not applicable”), after washing dishes (with an additional answer “not applicable”), and after doing laundry (with an additional answer “not applicable”). The circumstances of washing hands were consistent with the general recommendations provided by the WHO [49,50], CDC [22,51,52], and UNICEF [53].

Specific steps were distinguished within the handwashing procedure and, for each of them, the respondents were asked to clarify whether they follow this step or not (close-ended question), with categories, as follows: always, sometimes, never. For some steps, there was an additional answer indicating that this step is not applicable for the respondents. The steps of handwashing procedure were listed, as follows: folding sleeves (with an additional answer “not applicable”), removing watch and bracelets, as well as rings (both with an additional answer “not applicable”), using soap and warm water, soaking hands before using soap (with an additional answer “not applicable”, as they do not use soap), careful making soap lather on whole hands (with an additional answer “not applicable”, as they do not use soap), turning the faucet off with hand (being the only reverse question, as the faucet should not be turned off with bare had), and drying hands with towel. The handwashing procedure was formed on the basis of recommendations provided by WHO [49,50], CDC [22,51,52], and UNICEF [53].

### 2.3. Statistical Analysis

The study participants were compared between sub-groups stratified by region, based on the following traits:

- COVID-19 morbidity in the region, assessed based on the number of the COVID-19 cases in the voivodeships in Poland in April 2020 [41]—the voivodeships were divided into groups of low COVID-19 morbidity (10 voivodeships attributed to less than 30% of total number of COVID-19 cases in Poland) and high COVID-19 morbidity (six voivodeships attributed to more than 70% of total number of COVID-19 cases in Poland). The cumulative number of COVID-19 cases in each voivodeship classified as low COVID-19 morbidity was, in April 2020, lower than 150, 500, and 750 for the beginning, the middle, and end of the month, respectively [41];
- status of the region, assessed based on the gross domestic product (GDP) in the voivodeship, while the data by the Eurostat were used [54]—the voivodeships were divided into groups of low GDP (13 voivodeships) and high GDP (3 voivodeships). The low GDP was interpreted as 24–73% in purchasing power standard and high GDP was interpreted as 73–624% in purchasing power standard [54];
- environment (rural/urban), assessed based on the size of the town, as classified into groups of villages and small towns, medium cities, big cities—assessed based on the data of Polish Central Statistical Office [55]. The villages and small cities were defined as ones having < 20,000 inhabitants, medium cities—having 20,000–100,000 inhabitants, and big cities—having > 100,000 inhabitants [55].

The statistical analysis was based on the comparison of the share of groups, which was conducted using the chi^2^ test. The level of *p* ≤ 0.05 was interpreted as a significant difference between the compared groups. The statistical analysis was conducted while using Statgraphics Plus for Windows 5.1 (Statgraphics Technologies Inc., The Plains, VA, USA).

## 3. Results

### 3.1. Analysis of Hand Hygiene Behaviors in a National Representative Sample of Polish Adolescents in Regions Stratified by COVID-19 Morbidity

Table 2 presents the declared circumstances of washing hands that are associated with meals and personal hygiene, on the basis of the Handwashing Habits Questionnaire [47], in the studied national sample of adolescents, in regions stratified by COVID-19 morbidity. For the majority of assessed circumstances that are associated with meals, including situations before meals (*p* = 0.0196), after meals (*p* = 0.0041), and after preparing meals (*p* = 0.0297), the share of respondents declaring always washing their hands was significantly higher among adolescents from the voivodeships with low COVID-19 morbidity, when comparing to adolescents from the voivodeships with high COVID-19 morbidity. For the majority of assessed circumstances that are associated with personal hygiene, including situations before using the restroom (*p* = 0.0068), after using the restroom (*p* = 0.0014), and after combing their hair (*p* = 0.0298), the share of respondents declaring always washing their hands was significantly higher among the adolescents from the voivodeships with low COVID-19 morbidity, when comparing to adolescents from the voivodeships with high COVID-19 morbidity.

Table 3 presents the declared circumstances of washing hands that are associated with leaving home and socializing, on the basis of the Handwashing Habits Questionnaire [47], in the studied national sample of adolescents, in regions stratified by COVID-19 morbidity. For some of assessed circumstances that are associated with socializing, including handshaking (*p* = 0.0373), after touching animals (*p* = 0.0007), and after contacting babies (*p* = 0.0278), the share of respondents declaring always washing their hands was significantly higher among adolescents from the voivodeships with low COVID-19 morbidity, when comparing to adolescents from the voivodeships with high COVID-19 morbidity.

Table 4 presents the declared circumstances of washing hands that are associated with health and household chores, on the basis of the Handwashing Habits Questionnaire [47], in the studied national sample of adolescents, in regions stratified by COVID-19 morbidity. For some of assessed circumstances that are associated with health, including after blowing nose (*p* = 0.0435), and after touching sick people (*p* = 0.0351), the share of respondents declaring always washing their hands was significantly higher among adolescents from the voivodeships with low COVID-19 morbidity, when comparing to adolescents from the voivodeships with high COVID-19 morbidity. For some of assessed circumstances that are associated with household chores, including after cleaning home (*p* = 0.0234), the share of respondents declaring always washing their hands was significantly higher among adolescents from the voivodeships with low COVID-19 morbidity, when comparing to adolescents from the voivodeships with high COVID-19 morbidity.

Table 5 presents the declared handwashing procedure in the studied national sample of adolescents, in regions stratified by COVID-19 morbidity. For some of assessed steps of procedure, including removing watch and bracelets (*p* = 0.0052), removing rings before or during handwashing (*p* = 0.0318), and drying hands with towel (*p* = 0.0031), the share of respondents declaring including them always for washing their hands was significantly higher among adolescents from the voivodeships with low COVID-19 morbidity, when comparing to adolescents from the voivodeships with high COVID-19 morbidity.

### 3.2. Analysis of Hand Hygiene Behaviors in a National Representative Sample of Polish Adolescents in Regions Stratified by Gross Domestic Product (GDP)

Table 6 presents the declared circumstances of washing hands that are associated with meals and personal hygiene, on the basis of the Handwashing Habits Questionnaire [47], in the studied national sample of adolescents, in regions stratified by Gross Domestic Product (GDP). There were no differences observed for the assessed circumstances associated with meals among adolescents from the voivodeships with high and low GDP. For washing hands before using the restroom (*p* = 0.0355), the share of respondents declaring always washing their hands was significantly higher among adolescents from the voivodeships with high GDP, when comparing to adolescents from the voivodeships with low GDP.

Table 7 presents the declared circumstances of washing hands that are associated with leaving home and socializing, on the basis of the Handwashing Habits Questionnaire [47], in the studied national sample of adolescents, in regions stratified by Gross Domestic Product (GDP). For situation when hands are visibly soiled (*p* = 0.0314), the share of respondents declaring always washing their hands was significantly higher among adolescents from the voivodeships with low GDP, when comparing to adolescents from the voivodeships with high GDP. There were no differences observed in the assessed circumstances associated with leaving home among adolescents from the voivodeships with low and high GDP.

Table 8 presents the declared circumstances of washing hands that are associated with health and household chores, on the basis of the Handwashing Habits Questionnaire [47], in the studied national sample of adolescents, in regions stratified by COVID-19 morbidity. For situation after touching sick people (*p* = 0.0106), the share of respondents declaring always washing their hands was significantly higher among adolescents from the voivodeships with low GDP, when comparing to adolescents from the voivodeships with high GDP. For situation after cleaning their home (*p* = 0.0485), the share of respondents declaring always washing their hands was significantly higher among adolescents from the voivodeships with low GDP, when comparing to adolescents from the voivodeships with high GDP.

Supplementary Table S1 presents the declared handwashing procedure in the studied national sample of adolescents, in regions stratified by Gross Domestic Product (GDP). There were no differences observed in the declared handwashing procedure among adolescents from the voivodeships with low and high GDP.

### 3.3. Analysis of Hand Hygiene Behaviors in A National Representative Sample of Polish Adolescents in Regions Stratified by the Size of the Town

Table 9 presents the declared circumstances of washing hands that are associated with meals and personal hygiene, on the basis of the Handwashing Habits Questionnaire [47], in the studied national sample of adolescents, in regions stratified by the size of the town. For some of the assessed circumstances that are associated with meals, including situations before meals (*p* = 0.0027), after meals (*p* = 0.0020), the share of respondents declaring always washing their hands was significantly higher among adolescents from villages and small towns, when comparing to adolescents from medium and big cities. For the majority of assessed circumstances that are associated with personal hygiene, including situations before using the restroom (*p* = 0.0000), after waking up in the morning (*p* = 0.0430), and after combing their hair (*p* = 0.0000), the share of respondents declaring always washing their hands was significantly higher among adolescents from villages and small towns, when comparing to adolescents from medium and big cities.

Table 10 presents the declared circumstances of washing hands that are associated with leaving home and socializing, on the basis of the Handwashing Habits Questionnaire [47], in the studied national sample of adolescents, in regions stratified by the size of the town. There were no differences observed for assessed circumstances associated with leaving home among adolescents from villages and small towns, medium, and big cities. For some of assessed circumstances that are associated with socializing, including after handshaking (*p* = 0.0004), after touching animals (*p* = 0.0000), and after handling animal food (*p* = 0.0015), the share of respondents declaring always washing their hands was significantly higher among adolescents from villages and small towns, when comparing to medium and big cities.

Table 11 presents the declared circumstances of washing hands that are associated with health and household chores, on the basis of the Handwashing Habits Questionnaire [47], in the studied national sample of adolescents, in regions stratified by the size of the town. For the majority of assessed circumstances that are associated with health, including blowing nose (*p* = 0.0000), after sneezing (*p* = 0.0001), after coughing (*p* = 0.0001), and before touching sick people (*p* = 0.0260), the share of respondents declaring always washing their hands was significantly higher among adolescents from villages and small towns, when comparing to adults from medium and big cities. There were no differences observed in the assessed circumstances that are associated with household chores among adolescents from villages and small towns, medium, and big cities.

Table 12 presents the declared handwashing procedure in the studied national sample of adolescents, in regions stratified by the size of the town. For some of the assessed steps of procedure, including folding sleeves (*p* = 0.0063) and removing watch and bracelet (*p* = 0.0218), the share of respondents declaring including them always for washing their hands was significantly higher among adolescents from villages and small towns, comparing to adolescents from medium and big cities. The declared circumstances of washing hands, on the basis of the Handwashing Habits Questionnaire [47] and the declared handwashing procedure, in the studied national sample of adolescents in regions stratified by COVID-19 morbidity, Gross Domestic Product (GDP), and the size of the town are presented in Supplementary Tables S2 and S3, respectively.

## 4. Discussion

### 4.1. Analysis of Hand Hygiene Behaviors in Regions Stratified by COVID-19 Morbidity

In the period of April 2020, when presented study was conducted, morbidity in different regions of Poland was diversified, as there were voivodeships with a low or very low number of confirmed COVID-19 cases (10 voivodeships, with the lowest number in Lubusz voivodeship—lower than 100 cases eight weeks since the first confirmed case in Poland), while others were seriously affected (six voivodeships, with the highest number of cases in Masovian voivodeship—almost 2500 cases during the eight weeks since the first confirmed case in Poland) [41]. Among voivodeships in which a high number of confirmed cases was observed, there were Masovian, Silesian, Lower Silesian, and Lesser Poland voivodeships, which are the most populous regions in Poland [56]. At the same time, less populous voivodeships, such as Lublin, Podlaskie, and Lubusz voivodeships [56], were characterized by a lower number of COVID-19 cases. Therefore, it may be supposed that the virus spreading in specific regions of Poland may be partly dependent on the population density, as such an observation was also indicated for other areas in other countries [57,58]. It may be associated with the fact that keeping appropriate distance becomes more difficult in highly populated areas [58]. However, it does not fully explain the differences in COVID-19 morbidity, as, even in Poland, the differences of population density between voivodeships are not so significant to cause such important differences in a number of cases, while some voivodeships that are characterized by similar population density sometimes differ significantly in regard to COVID-19 morbidity (as Łódź voivodeship had significantly more COVID-19 cases than Pomeranian voivodeship [41,56]).

It is indicated that fear and anxiety towards COVID-19 infection may have a substantial influence on the adherence to the hygienic practices, as, in the study of Apisarnthanarak et al. [59], it was showed that most of healthcare workers experienced the feeling of fear and anxiety and these emotions resulted in more appropriate preventive practices, including handwashing. Similarly, in the study of Harper et al. [60], it was revealed that fear of COVID-19 infection was the only predictor of beneficial change of behaviors, such as improved hand hygiene and social distancing. Additionally, the study of Israel et al. [61] showed that the average hand hygiene compliance rate increased substantially in the group of hospital workers from January 2020 to April 2020 (46% vs. 89%), as the pandemic progressed. Therefore, it could have been assumed that, if the COVID-19 pandemic had influenced hand hygiene behaviors, they would have been more beneficial in the case of more affected voivodeships. Meanwhile, in the studied population, the observed situation was different—with more beneficial hand hygiene behaviors in voivodeships being less affected. Such a relation may suggest the reverse association, namely the influence of hand hygiene on the COVID-19 morbidity. It should be taken into consideration that the COVID-19 infection may occur when a person touches a surface that is contaminated with SARS-CoV-2, and after that hands have contact with mouth, nose, or eyes [62], so effective handwashing with water and soap is recommended [7]. It corresponds with the fact that, in the presented study, adolescents from the voivodeships with low COVID-19 morbidity were more likely to always wash their hands in specific circumstances associated with meals, such as before meals, after meals, and after preparing meals, as well as being associated with personal hygiene, such as before using the restroom, after using the restroom, and after combing their hair.

Generally, various factors, also other than those that are associated with health state and comorbidities, may influence an increase of the number of COVID-19 cases diagnosed, including family habits, age distribution, and societal customs [63]. It is hypothesized that all of the above-mentioned factors probably contributed to such high rates of COVID-19 infections that were observed in Italy. Traditionally, in Italy and other Mediterranean countries, relationships between family members are very close with frequent physical contact, which makes intergenerational contacts important determinants of the high infection rate in Italy [63]. Another important factor, not only for Italy, but also for Germany, which also reported a high number of diagnosed COVID-19 cases, is age distribution. The median age of population in Italy is 46.7 years, being the highest in Europe, and Germany has the second most aged population in Europe with the median age of 46.0 years, while indicated countries are characterized by the lowest share of young citizens (younger than 14 years old) [64]. Therefore, high rates of COVID-19 infections in these countries may be partially explained by the higher age of their inhabitants. In Poland, a voivodeship with the highest median age is Opole voivodeship, being characterized by the median of 43.1 years; however, it does not correspond with the number of COVID-19 cases, as, in this voivodeship, a low number of cases was stated [41,65]. On the other hand, some seriously affected voivodeships, such as Łódź, Silesian and Lower Silesian voivodeships are characterized by one of the highest median age, which, for these regions, may partially explain the high number of COVID-19 cases diagnosed [41,65]. Therefore, it seems that age distribution may not be a crucial factor influencing COVID-19 morbidity, but it may play a role among other influencing factors.

### 4.2. Analysis of Hand Hygiene Behaviors in Regions Stratified by Gross Domestic Product (GDP)

It is pointed out that economic and social factors may also influence the dynamics of the transmission of infectious diseases [66]. Based on the presented results, it may be supposed that in the conducted study, among the most important factors influencing morbidity in the regions of Poland is washing hands, while the socioeconomic status of the region may be treated as a possible interfering factor. In the our own presented study, it was found that adolescents from the regions characterized by higher GDP were more likely to always wash their hands before using the restroom, but they were also less likely to always wash their hands when hands are visibly soiled, after touching sick people, and after cleaning their home, when comparing to adolescents from the regions that are characterized by lower GDP.

The association between economic status and hygienic behaviors was mainly studied in low-income countries, while, in developed countries, this issue is rarely analyzed. It is stated that in low-income countries, socio-economic factors, including education of household head and respondent, water availability, and access to media have a strong positive association with hand washing with soap [67]. At the same time, handwashing indicators are common among households with higher socioeconomic status [68].

However, for an association between GDP and resultant COVID-19 cases, similarly as in our own presented study, other authors observed contradictory results. In the study of You et al. [69] analyzing the distribution of COVID-19 morbidity rates in Wuhan, China, it was stated that higher GDP per unit of land area was associated with a decreased COVID-19 morbidity rate. Moreover, a meta-regression of data from fifty biggest cities in United States indicated that the socioeconomic status of inhabitants may affect COVID-19 prevalence, as well as case fatality, because COVID-19 prevalence was significantly associated with the poverty rate [70]. However, in the study of Sarmadi et al. [71], assessing the global distribution of COVID-19, depending on demographic and environmental factors, it was found that countries with higher GDP had a higher proportion of COVID-19 cases to population. While taking this into account, it may be indicated that GDP for the region did not present unambiguous association with hand hygiene practices and resultant COVID-19 morbidity.

### 4.3. Analysis of Hand Hygiene Behaviors in Regions Stratified by the Size of the Town

Based on the presented results, it may be supposed that, in the conducted study, an association between morbidity in the regions of Poland and hand-washing habits was interfered by the rural/urban environment. In the presented study, it was observed that adolescents from villages and small towns generally washed their hands significantly more often than adolescents from medium and big cities in specific circumstances. However, such results are inconsistent with those that were obtained by other authors, because, usually, better hand hygiene practices are indicated for inhabitants of urban areas.

In study undertaken by Mane et al. [72], which was conducted among school students from rural and urban areas in India, it was stated that children from urban areas were more likely to use water and soap for hand washing, when comparing to children from rural areas. A study by Khan et al. [73], which was carried out in a group of children caretakers, indicated that caregivers from urban areas were more likely to wash their hands with water and soap in some circumstances, such as before preparing food, after using toilet, or after returning from outside, than caregivers from rural areas.

However, similarly to the influence of GDP, there are no such studies conducted in developed countries, so the indicated results may be incomparable if the characteristics of countries differ. Moreover, the period of COVID-19 pandemic may change the described pattern, as big cities may no longer be a preferable place to live, while people are moving from major places of concentration, into less densely populated communities [74]. At the same time, the rapid migrations of people to cities can lead to overcrowding, which can generate slums or shanty towns [75].

In summary, it must be emphasized that understanding the distribution of COVID-19 morbidity, depending on hand hygiene behaviors, as well as socioeconomic status and rural/urban setting of the affected regions, can result in more effective control of the COVID-19 spreading. The obtained information may be helpful in creating more successful educational campaigns concerning hand hygiene behaviors, targeted at specific regions that are affected by the COVID-19 pandemic, while taking potential interfering factors into consideration, such as socioeconomic status and rural/urban setting.

## 5. Conclusions

In regions of low COVID-19 morbidity, a higher share of adolescents than in regions of high morbidity declared washing their hands in various circumstances that are associated with meals, personal hygiene, leaving home, socializing, health, and household chores. Similarly, for the assessed steps of handwashing procedure, in regions of low COVID-19 morbidity, a higher share of adolescents included them to their daily handwashing, than in regions of high morbidity. For the comparison in regions stratified by GDP, the differences were only minor and inconsistent, while, for the comparison in place of residence stratified by number of residents in city, there were some minor differences, indicating better hand hygiene behaviors in the case of villages and small towns when compared with medium and large cities. It may be concluded that, in a population-based sample of Polish adolescents, individuals from regions of low COVID-19 morbidity presented more beneficial hand hygiene habits than those from regions of high COVID-19 morbidity.

## Figures and Tables

**Table 1:** The cumulative number of the COVID-19 cases in the voivodeships in Poland in April 2020.

Voivodeship		1st April	8th April	15th April	22nd April	29th April
Voivodeships of low COVID-19 morbidity	Podlaskie	44	149	259	333	367
	Lubusz	47	66	79	84	87
	Holy Cross	55	127	170	242	266
	Pomeranian	56	143	179	227	422
	Warmian-Masurian	59	92	125	145	146
	Opole	73	116	219	316	386
	Kuyavian-Pomeranian	76	277	359	452	537
	West Pomeranian	76	165	217	284	380
	Subcarpathian	100	182	243	271	328
	Lublin	132	199	270	332	359
Voivodeships of high COVID-19 morbidity	Greater Poland	158	394	785	1097	1399
	Lesser Poland	209	398	561	726	863
	Łódź	221	400	572	750	904
	Silesian	272	644	988	1580	2080
	Lower Silesian	298	539	561	1136	1500
	Masovian	544	1314	1792	2059	2391
Total for Poland		2420	5205	7582	10,034	12,415

**Table 2:** The declared circumstances of washing hands associated with meals and personal hygiene, on the basis of the Handwashing Habits Questionnaire, in the studied national sample of adolescents, in regions stratified by COVID-19 morbidity (comparison conducted while using chi^2^ test).

Characteristics		Low COVID-19 Morbidity in Region	High COVID-19 Morbidity in Region	*p*
**Before meals**	Always	445 (72.2%)	394 (65.0%)	0.0196
	Sometimes	156 (25.3%)	189 (31.2%)	
	Never	15 (2.4%)	23 (3.8%)	
**After meals**	Always	185 (30.0%)	164 (27.1%)	0.0041
	Sometimes	358 (58.1%)	329 (54.3%)	
	Never	73 (11.9%)	113 (18.6%)	
**Before preparing meals**	Always	519 (84.3%)	495 (81.7%)	0.7453
	Sometimes	48 (7.8%)	51 (8.4%)	
	Never	6 (1.0%)	4 (0.7%)	
	Not applicable *	43 (7.0%)	56 (9.2%)	
**After preparing meals**	Always	432 (70.1%)	374 (61.7%)	0.0297
	Sometimes	126 (20.5%)	155 (25.6%)	
	Never	17 (2.8%)	21 (3.5%)	
	Not applicable *	41 (6.7%)	56 (9.2%)	
**Before using the restroom**	Always	107 (17.4%)	68 (11.2%)	0.0068
	Sometimes	219 (35.6%)	244 (40.3%)	
	Never	290 (47.1%)	294 (48.5%)	
**After using the restroom**	Always	593 (96.3%)	571 (94.2%)	0.0014
	Sometimes	15 (2.4%)	34 (5.6%)	
	Never	8 (1.3%)	1 (0.2%)	
**Before going to bed**	Always	265 (43.0%)	256 (42.2%)	0.7722
	Sometimes	208 (33.8%)	216 (35.6%)	
	Never	143 (23.2%)	134 (22.1%)	
**After waking up in the morning**	Always	272 (44.2%)	265 (43.7%)	0.2235
	Sometimes	212 (34.4%)	188 (31.0%)	
	Never	132 (21.4%)	153 (25.2%)	
**After combing their hair**	Always	158 (25.6%)	115 (19.0%)	0.0298
	Sometimes	196 (31.8%)	203 (33.5%)	
	Never	205 (33.3%)	220 (36.3%)	
	Not applicable *	57 (9.3%)	68 (11.2%)	

* not included to statistical analysis.

**Table 3:** The declared circumstances of washing hands associated with leaving home and socializing, on the basis of the Handwashing Habits Questionnaire, in the studied national sample of adolescents, in regions stratified by COVID-19 morbidity (comparison conducted while using the chi^2^ test).

Characteristics		Low COVID-19 Morbidity in Region	High COVID-19 Morbidity in Region	*p*
**After coming back home**	Always	536 (87.0%)	538 (88.8%)	0.5363
	Sometimes	75 (12.2%)	62 (10.2%)	
	Never	5 (0.8%)	6 (1.0%)	
**After using public transportation**	Always	473 (76.8%)	470 (77.6%)	0.8816
	Sometimes	107 (17.4%)	99 (16.3%)	
	Never	36 (5.8%)	37 (6.1%)	
**After money exchange**	Always	334 (54.2%)	312 (51.5%)	0.5120
	Sometimes	171 (27.8%)	186 (30.7%)	
	Never	111 (18.0%)	108 (17.8%)	
**When hands are visibly soiled**	Always	528 (85.7%)	487 (80.4%)	0.1236
	Sometimes	66 (10.7%)	84 (13.9%)	
	Never	22 (3.6%)	35 (5.8%)	
**After handshaking**	Always	297 (48.2%)	248 (40.9%)	0.0373
	Sometimes	228 (37.0%)	255 (42.1%)	
	Never	91 (14.8%)	103 (17.0%)	
**After touching animals**	Always	317 (51.5%)	253 (41.7%)	0.0007
	Sometimes	160 (26%)	181 (29.9%)	
	Never	27 (4.4%)	49 (8.1%)	
	Not applicable *	112 (18.2%)	123 (20.3%)	
**After handling animal waste**	Always	432 (70.1%)	416 (68.6%)	0.8981
	Sometimes	39 (6.3%)	39 (6.4%)	
	Never	17 (2.8%)	14 (2.3%)	
	Not applicable *	128 (20.8%)	137 (22.6%)	
**After handling animal food**	Always	336 (54.5%)	305 (50.3%)	0.3763
	Sometimes	119 (19.3%)	129 (21.3%)	
	Never	37 (6.0%)	42 (6.9%)	
	Not applicable *	124 (20.1%)	130 (21.5%)	
**After contacting babies**	Always	113 (18.3%)	72 (11.9%)	0.0278
	Sometimes	101 (16.4%)	98 (16.2%)	
	Never	38 (6.2%)	46 (7.6%)	
	Not applicable *	364 (59.1%)	390 (64.4%)	
**After changing diapers**	Always	160 (26.0%)	101 (16.7%)	0.2487
	Sometimes	21 (3.4%)	15 (2.5%)	
	Never	9 (1.5%)	12 (2.0%)	
	Not applicable *	426 (69.2%)	478 (78.9%)	

* not included to statistical analysis.

**Table 4:** The declared circumstances of washing hands associated with health and household chores, on the basis of the Handwashing Habits Questionnaire, in the studied national sample of adolescents, in regions stratified by COVID-19 morbidity (comparison conducted while using chi^2^ test).

Characteristics		Low COVID-19 Morbidity in Region	High COVID-19 Morbidity in Region	*p*
**After blowing nose**	Always	250 (40.6%)	204 (33.7%)	0.0435
	Sometimes	260 (42.2%)	285 (47.0%)	
	Never	106 (17.2%)	117 (19.3%)	
**After sneezing**	Always	280 (45.5%)	245 (40.4%)	0.1080
	Sometimes	253 (41.1%)	258 (42.6%)	
	Never	83 (13.5%)	103 (17.0%)	
**After coughing**	Always	257 (41.7%)	222 (36.6%)	0.1831
	Sometimes	253 (41.1%)	267 (44.1%)	
	Never	106 (17.2%)	117 (19.3%)	
**Before touching sick people**	Always	271 (44.0%)	255 (42.1%)	0.1074
	Sometimes	202 (32.8%)	179 (29.5%)	
	Never	143 (23.2%)	172 (28.4%)	
**After touching sick people**	Always	528 (85.7%)	487 (80.4%)	0.0351
	Sometimes	66 (10.7%)	84 (13.9%)	
	Never	22 (3.6%)	35 (5.8%)	
**After touching garbage**	Always	474 (76.9%)	447 (73.8%)	0.8142
	Sometimes	81 (13.1%)	80 (13.2%)	
	Never	17 (2.8%)	13 (2.1%)	
	Not applicable *	44 (7.1%)	66 (10.9%)	
**After cleaning their home**	Always	430 (69.8%)	387 (63.9%)	0.0234
	Sometimes	148 (24.0%)	181 (29.9%)	
	Never	27 (4.4%)	17 (2.8%)	
	Not applicable *	11 (1.8%)	21 (3.5%)	
**After washing dishes**	Always	392 (63.6%)	356 (58.7%)	0.0741
	Sometimes	91 (14.8%)	112 (18.5%)	
	Never	49 (8.0%)	61 (10.1%)	
	Not applicable *	84 (13.6%)	77 (12.7%)	
**After doing laundry**	Always	182 (29.5%)	160 (26.4%)	0.1720
	Sometimes	176 (28.6%)	150 (24.8%)	
	Never	100 (16.2%)	116 (19.1%)	
	Not applicable *	158 (25.6%)	180 (29.7%)	

* not included to statistical analysis.

**Table 5:** The declared handwashing procedure in the studied national sample of adolescents, in regions stratified by COVID-19 morbidity (comparison conducted while using chi^2^ test).

Characteristics		Low COVID-19 Morbidity in Region	High COVID-19 Morbidity in Region	*p*
**Folding sleeves**	Always	332 (53.9%)	289 (47.7%)	0.0667
	Sometimes	116 (18.8%)	133 (21.9%)	
	Never	21 (3.4%)	30 (5.0%)	
	Not applicable *	147 (23.9%)	154 (25.4%)	
**Removing watch and bracelets**	Always	92 (14.9%)	68 (11.2%)	0.0052
	Sometimes	123 (20.0%)	93 (15.3%)	
	Never	45 (7.3%)	68 (11.2%)	
	Not applicable *	356 (57.8%)	377 (62.2%)	
**Removing rings before or during handwashing**	Always	68 (11.0%)	54 (8.9%)	0.0318
	Sometimes	48 (7.8%)	33 (5.4%)	
	Never	40 (6.5%)	57 (9.4%)	
	Not applicable *	460 (74.7%)	462 (76.2%)	
**Using soap**	Always	580 (94.2%)	566 (93.4%)	0.7258
	Sometimes	33 (5.4%)	38 (6.3%)	
	Never	3 (0.5%)	2 (0.3%)	
**Using warm water**	Always	381 (61.9%)	383 (63.2%)	0.6845
	Sometimes	222 (36.0%)	207 (34.2%)	
	Never	13 (2.1%)	16 (2.6%)	
**Soaking hands before using soap**	Always	441 (71.6%)	438 (72.3%)	0.3727
	Sometimes	102 (16.6%)	110 (18.2%)	
	Never	72 (11.7%)	57 (9.4%)	
	Not applicable *	1 (0.2%)	1 (0.2%)	
**Careful making soap lather on whole hands**	Always	376 (61.0%)	360 (59.4%)	0.8098
	Sometimes	214 (34.7%)	216 (35.6%)	
	Never	26 (4.3%)	29 (4.8%)	
	Not applicable *	0 (0%)	1 (0.2%)	
**Turning the faucet off with hand**	Always	314 (51.0%)	302 (49.8%)	0.7012
	Sometimes	170 (27.6%)	162 (26.7%)	
	Never	132 (21.4%)	142 (23.4%)	
**Drying hands with towel**	Always	523 (84.9%)	466 (76.9%)	0.0031
	Sometimes	77 (12.5%)	122 (20.1%)	
	Never	16 (2.6%)	18 (2.9%)	

* not included to statistical analysis.

**Table 6:** The declared circumstances of washing hands associated with meals and personal hygiene, on the basis of the Handwashing Habits Questionnaire, in the studied national sample of adolescents, in regions stratified by Gross Domestic Product (GDP) (comparison conducted while using chi^2^ test).

Characteristics		Low GDP in Region	High GDP in Region	*p*
**Before meals**	Always	650 (67.9%)	189 (71.3%)	0.1949
	Sometimes	273 (28.5%)	72 (27.2%)	
	Never	34 (3.6%)	4 (1.5%)	
**After meals**	Always	264 (27.6%)	85 (32.1%)	0.2452
	Sometimes	541 (56.5%)	146 (55.1%)	
	Never	152 (15.9%)	34 (12.8%)	
**Before preparing meals**	Always	802 (83.8%)	212 (80.0%)	0.1935
	Sometimes	78 (8.2%)	21 (7.9%)	
	Never	8 (0.8%)	2 (0.8%)	
	Not applicable *	69 (7.2%)	30 (11.3%)	
**After preparing meals**	Always	642 (67.1%)	164 (61.9%)	0.4502
	Sometimes	216 (22.6%)	65 (24.5%)	
	Never	32 (3.3%)	6 (2.3%)	
	Not applicable *	67 (7.0%)	30 (11.3%)	
**Before using the restroom**	Always	136 (14.2%)	39 (14.7%)	0.0355
	Sometimes	346 (36.1%)	117 (44.2%)	
	Never	475 (48.7%)	109 (41.1%)	
**After using the restroom**	Always	910 (95.1%)	254 (95.8%)	0.2834
	Sometimes	38 (4.0%)	11 (4.2%)	
	Never	9 (0.9%)	0 (0.0%)	
**Before going to bed**	Always	408 (42.6%)	113 (42.6%)	0.6373
	Sometimes	327 (34.2%)	97 (36.6%)	
	Never	222 (23.2%)	55 (20.8%)	
**After waking up in the morning**	Always	406 (42.4%)	131 (49.4%)	0.1249
	Sometimes	321 (33.6%)	79 (29.8%)	
	Never	230 (24.0%)	55 (20.8%)	
**After combing their hair**	Always	208 (21.8%)	65 (24.5%)	0.4800
	Sometimes	311 (32.5%)	88 (33.2%)	
	Never	340 (35.5%)	85 (32.1%)	
	Not applicable *	98 (10.2%)	27 (10.2%)	

* not included to statistical analysis.

**Table 7:** The declared circumstances of washing hands associated with leaving home and socializing, on the basis of the Handwashing Habits Questionnaire, in the studied national sample of adolescents, in regions stratified by Gross Domestic Product (GDP) (comparison conducted while using chi^2^ test).

Characteristics		Low GDP in Region	High GDP in Region	*p*
**After coming back home**	Always	837 (87.5%)	237 (89.5%)	0.5361
	Sometimes	112 (11.7%)	25 (9.4%)	
	Never	8 (0.8%)	3 (1.1%)	
**After using public transportation**	Always	737 (77.0%)	206 (77.7%)	0.6774
	Sometimes	165 (17.2%)	41 (15.5%)	
	Never	55 (5.8%)	18 (6.8%)	
**After money exchange**	Always	521 (54.5%)	125 (47.2%)	0.1092
	Sometimes	271 (28.3%)	86 (32.4%)	
	Never	165 (17.2%)	54 (20.4%)	
**When hands are visibly soiled**	Always	931 (97.3%)	249 (94.0%)	0.0314
	Sometimes	19 (2.0%)	12 (4.5%)	
	Never	7 (0.7%)	4 (1.5%)	
**After handshaking**	Always	426 (44.5%)	119 (44.9%)	0.9656
	Sometimes	380 (39.7%)	103 (38.9%)	
	Never	151 (15.8%)	43 (16.2%)	
**After touching animals**	Always	452 (47.3%)	118 (44.5%)	0.1397
	Sometimes	264 (27.6%)	77 (29.1%)	
	Never	52 (5.4%)	24 (9.1%)	
	Not applicable *	189 (19.7%)	46 (17.3%)	
**After handling animal waste**	Always	660 (69.0%)	188 (70.9%)	0.9319
	Sometimes	61 (6.4%)	17 (6.4%)	
	Never	25 (2.6%)	6 (2.3%)	
	Not applicable *	211 (22.0%)	54 (20.4%)	
**After handling animal food**	Always	510 (53.3%)	131 (49.4%)	0.1299
	Sometimes	181 (18.9%)	67 (25.3%)	
	Never	61 (6.4%)	18 (6.8%)	
	Not applicable *	205 (21.4%)	49 (18.5%)	
**After contacting babies**	Always	155 (16.2%)	30 (11.3%)	0.2844
	Sometimes	154 (16.1%)	45 (17.0%)	
	Never	68 (7.1%)	16 (6.0%)	
	Not applicable *	580 (60.6%)	174 (65.7%)	
**After changing diapers**	Always	209 (21.8%)	52 (19.6%)	0.2475
	Sometimes	32 (3.3%)	4 (1.5%)	
	Never	19 (2.0%)	2 (0.8%)	
	Not applicable *	697 (72.9%)	207 (78.1%)	

* not included to statistical analysis.

**Table 8:** The declared circumstances of washing hands associated with health and household chores, on the basis of the Handwashing Habits Questionnaire, in the studied national sample of adolescents, in regions stratified by Gross Domestic Product (GDP) (comparison conducted while using chi^2^ test).

Characteristics		Low GDP in Region	High GDP in Region	*p*
**After blowing nose**	Always	346 (36.1%)	108 (40.8%)	0.3505
	Sometimes	436 (45.6%)	109 (41.1%)	
	Never	175 (18.3%)	48 (18.1%)	
**After sneezing**	Always	405 (42.3%)	120 (45.3%)	0.1555
	Sometimes	413 (43.2%)	98 (37.0%)	
	Never	139 (14.5%)	47 (17.7%)	
**After coughing**	Always	370 (38.7%)	109 (41.1%)	0.7012
	Sometimes	413 (43.1%)	107 (40.4%)	
	Never	174 (18.2%)	49 (18.5%)	
**Before touching sick people**	Always	416 (43.5%)	110 (41.5%)	0.6335
	Sometimes	292 (30.5%)	89 (33.6%)	
	Never	249 (26.0%)	66 (24.9%)	
**After touching sick people**	Always	811 (84.7%)	204 (77.0%)	0.0106
	Sometimes	107 (11.2%)	43 (16.2%)	
	Never	39 (4.1%)	18 (6.8%)	
**After touching garbage**	Always	719 (75.1%)	202 (76.3%)	0.8704
	Sometimes	130 (13.6%)	31 (11.7%)	
	Never	23 (2.4%)	7 (2.6%)	
	Not applicable *	85 (8.9%)	25 (9.4%)	
**After cleaning their home**	Always	656 (68.5%)	161 (60.8%)	0.0485
	Sometimes	243 (25.4%)	86 (32.4%)	
	Never	36 (3.8%)	8 (3.0%)	
	Not applicable *	22 (2.3%)	10 (3.8%)	
**After washing dishes**	Always	591 (61.8%)	157 (59.2%)	0.8068
	Sometimes	157 (16.4%)	46 (17.4%)	
	Never	87 (9.1%)	23 (8.7%)	
	Not applicable *	122 (12.7%)	39 (14.7%)	
**After doing laundry**	Always	267 (27.9%)	75 (28.3%)	0.8065
	Sometimes	260 (27.2%)	66 (24.9%)	
	Never	173 (18.1%)	43 (16.2%)	
	Not applicable *	257 (26.8%)	81 (30.6%)	

* not included to statistical analysis.

**Table 9:** The declared circumstances of washing hands associated with meals and personal hygiene, on the basis of the Handwashing Habits Questionnaire, in the studied national sample of adolescents, in regions stratified by the size of the town (comparison conducted while using the chi^2^ test).

Characteristics		Villages and Small Towns	Medium Cities	Big Cities	*p*
**Before meals**	Always	172 (78.9%)	393 (68%)	274 (64.3%)	0.0027
	Sometimes	44 (20.2%)	167 (28.9%)	134 (31.5%)	
	Never	2 (0.9%)	18 (3.1%)	18 (4.2%)	
**After meals**	Always	78 (35.8%)	169 (29.2%)	102 (23.9%)	0.0020
	Sometimes	122 (56.0%)	318 (55.0%)	247 (58.0%)	
	Never	18 (8.3%)	91 (15.7%)	77 (18.1%)	
**Before preparing meals**	Always	189 (86.7%)	476 (82.4%)	349 (81.9%)	0.7148
	Sometimes	14 (6.4%)	46 (8%)	39 (9.2%)	
	Never	1 (0.5%)	5 (0.9%)	4 (0.9%)	
	Not applicable *	14 (6.4%)	51 (8.8%)	34 (8.0%)	
**After preparing meals**	Always	160 (73.4%)	366 (63.3%)	280 (65.7%)	0.0524
	Sometimes	43 (19.7%)	144 (24.9%)	94 (22.1%)	
	Never	2 (0.9%)	18 (3.1%)	18 (4.2%)	
	Not applicable *	13 (6.0%)	50 (8.7%)	34 (8.0%)	
**Before using the restroom**	Always	55 (25.2%)	74 (12.8%)	46 (10.8%)	0.0000
	Sometimes	79 (36.2%)	232 (40.1%)	152 (35.7%)	
	Never	84 (38.5%)	272 (47.1%)	228 (53.5%)	
**After using the restroom**	Always	210 (96.3%)	542 (93.8%)	412 (96.7%)	0.2063
	Sometimes	6 (2.8%)	31 (5.4%)	12 (2.8%)	
	Never	2 (0.9%)	5 (0.9%)	2 (0.5%)	
**Before going to bed**	Always	101 (46.3%)	234 (40.5%)	186 (43.7%)	0.1478
	Sometimes	80 (36.7%)	208 (36.0%)	136 (31.9%)	
	Never	37 (17.0%)	136 (23.5%)	104 (24.4%)	
**After waking up in the morning**	Always	111 (50.9%)	245 (42.4%)	181 (42.5%)	0.0430
	Sometimes	73 (33.5%)	190 (32.9%)	137 (32.2%)	
	Never	34 (15.6%)	143 (24.7%)	108 (25.4%)	
**After combing their hair**	Always	79 (36.2%)	127 (22%)	67 (15.7%)	0.0000
	Sometimes	60 (27.5%)	212 (36.7%)	127 (29.8%)	
	Never	61 (28%)	183 (31.7%)	181 (42.5%)	
	Not applicable *	18 (8.3%)	56 (9.7%)	51 (12.0%)	

* not included to statistical analysis.

**Table 10:** The declared circumstances of washing hands associated with leaving home and socializing, on the basis of the Handwashing Habits Questionnaire, in the studied national sample of adolescents, in regions stratified by the size of the town (comparison conducted while using chi^2^ test).

Characteristics		Villages and Small Towns	Medium Cities	Big Cities	*p*
**After coming back home**	Always	191 (87.6%)	506 (87.5%)	377 (88.5%)	0.5086
	Sometimes	24 (11%)	65 (11.2%)	48 (11.3%)	
	Never	3 (1.4%)	7 (1.2%)	1 (0.2%)	
**After using public transportation**	Always	167 (76.6%)	443 (76.6%)	333 (78.2%)	0.6704
	Sometimes	34 (15.6%)	100 (17.3%)	72 (16.9%)	
	Never	17 (7.8%)	35 (6.1%)	21 (4.9%)	
**After money exchange**	Always	131 (60.1%)	294 (50.9%)	221 (51.9%)	0.0839
	Sometimes	47 (21.6%)	178 (30.8%)	132 (31.0%)	
	Never	40 (18.3%)	106 (18.3%)	73 (17.1%)	
**When hands are visibly soiled**	Always	213 (97.7%)	552 (95.5%)	415 (97.4%)	0.0536
	Sometimes	4 (1.8%)	22 (3.8%)	5 (1.2%)	
	Never	1 (0.5%)	4 (0.7%)	6 (1.4%)	
**After handshaking**	Always	121 (55.5%)	237 (41.0%)	187 (43.9%)	0.0004
	Sometimes	67 (30.7%)	258 (44.6%)	158 (37.1%)	
	Never	30 (13.8%)	83 (14.4%)	81 (19.0%)	
**After touching animals**	Always	143 (65.6%)	271 (46.9%)	156 (36.6%)	0.0000
	Sometimes	39 (17.9%)	167 (28.9%)	135 (31.7%)	
	Never	7 (3.2%)	40 (6.9%)	29 (6.8%)	
	Not applicable *	29 (13.3%)	100 (17.3%)	106 (24.9%)	
**After handling animal waste**	Always	172 (78.9%)	403 (69.7%)	273 (64.1%)	0.6382
	Sometimes	12 (5.5%)	42 (7.3%)	24 (5.6%)	
	Never	4 (1.8%)	17 (2.9%)	10 (2.3%)	
	Not applicable *	30 (13.8%)	116 (20.1%)	119 (27.9%)	
**After handling animal food**	Always	144 (66.1%)	296 (51.2%)	201 (47.2%)	0.0015
	Sometimes	38 (17.4%)	136 (23.5%)	74 (17.4%)	
	Never	7 (3.2%)	36 (6.2%)	36 (8.5%)	
	Not applicable *	29 (13.3%)	110 (19%)	115 (27.0%)	
**After contacting babies**	Always	55 (25.2%)	87 (15.1%)	43 (10.1%)	0.2107
	Sometimes	40 (18.3%)	103 (17.8%)	56 (13.1%)	
	Never	17 (7.8%)	45 (7.8%)	22 (5.2%)	
	Not applicable *	106 (48.6%)	343 (59.3%)	305 (71.6%)	
**After changing diapers**	Always	79 (36.2%)	116 (20.1%)	66 (15.5%)	0.1877
	Sometimes	10 (4.6%)	22 (3.8%)	4 (0.9%)	
	Never	4 (1.8%)	10 (1.7%)	7 (1.6%)	
	Not applicable *	125 (57.3%)	430 (74.4%)	349 (81.9%)	

* not included to statistical analysis.

**Table 11:** The declared circumstances of washing hands associated with health and household chores, on the basis of the Handwashing Habits Questionnaire, in the studied national sample of adolescents, in regions stratified by Gross Domestic Product (GDP) (comparison conducted while using chi^2^ test).

Characteristics		Villages and Small Towns	Medium Cities	Big Cities	*p*
**After blowing nose**	Always	112 (51.4%)	209 (36.2%)	133 (31.2%)	0.0000
	Sometimes	77 (35.3%)	264 (45.7%)	204 (47.9%)	
	Never	29 (13.3%)	105 (18.2%)	89 (20.9%)	
**After sneezing**	Always	122 (56.0%)	246 (42.6%)	157 (36.9%)	0.0001
	Sometimes	70 (32.1%)	253 (43.8%)	188 (44.1%)	
	Never	26 (11.9%)	79 (13.7%)	81 (19.0%)	
**After coughing**	Always	110 (50.5%)	230 (39.8%)	139 (32.6%)	0.0001
	Sometimes	78 (35.8%)	253 (43.8%)	189 (44.4%)	
	Never	30 (13.8%)	95 (16.4%)	98 (23.0%)	
**Before touching sick people**	Always	108 (49.5%)	251 (43.4%)	167 (39.2%)	0.0260
	Sometimes	71 (32.6%)	171 (29.6%)	139 (32.6%)	
	Never	39 (17.9%)	156 (27.0%)	120 (28.2%)	
**After touching sick people**	Always	193 (88.5%)	472 (81.7%)	350 (82.2%)	0.0876
	Sometimes	15 (6.9%)	76 (13.1%)	59 (13.8%)	
	Never	10 (4.6%)	30 (5.2%)	17 (4.0%)	
**After touching garbage**	Always	178 (81.7%)	440 (76.1%)	303 (71.1%)	0.3727
	Sometimes	25 (11.5%)	70 (12.1%)	66 (15.5%)	
	Never	6 (2.8%)	14 (2.4%)	10 (2.3%)	
	Not applicable *	9 (4.1%)	54 (9.3%)	47 (11.0%)	
**After cleaning their home**	Always	160 (73.4%)	380 (65.7%)	277 (65.0%)	0.0582
	Sometimes	42 (19.3%)	166 (28.7%)	121 (28.4%)	
	Never	11 (5%)	18 (3.1%)	15 (3.5%)	
	Not applicable *	5 (2.3%)	14 (2.4%)	13 (3.1%)	
**After washing dishes**	Always	142 (65.1%)	344 (59.5%)	262 (61.5%)	0.1896
	Sometimes	36 (16.5%)	110 (19%)	57 (13.4%)	
	Never	16 (7.3%)	58 (10%)	36 (8.5%)	
	Not applicable *	24 (11%)	66 (11.4%)	71 (16.7%)	
**After doing laundry**	Always	84 (38.5%)	158 (27.3%)	100 (23.5%)	0.0531
	Sometimes	59 (27.1%)	168 (29.1%)	99 (23.2%)	
	Never	32 (14.7%)	110 (19.0%)	74 (17.4%)	
		43 (19.7%)	142 (24.6%)	153 (35.9%)	

* not included to statistical analysis.

**Table 12:** The declared handwashing procedure in the studied national sample of adolescents, in regions stratified by the size of the town (comparison conducted while using the chi^2^ test).

Characteristics		Villages and Small Towns	Medium Cities	Big Cities	*p*
**Folding sleeves**	Always	130 (59.6%)	307 (53.1%)	184 (43.2%)	0.0063
	Sometimes	38 (17.4%)	112 (19.4%)	99 (23.2%)	
	Never	4 (1.8%)	25 (4.3%)	22 (5.2%)	
	Not applicable *	46 (21.1%)	134 (23.2%)	121 (28.4%)	
**Removing watch and bracelets**	Always	41 (18.8%)	79 (13.7%)	40 (9.4%)	0.0218
	Sometimes	48 (22%)	94 (16.3%)	74 (17.4%)	
	Never	13 (6%)	60 (10.4%)	40 (9.4%)	
	Not applicable *	116 (53.2%)	345 (59.7%)	272 (63.8%)	
**Removing rings before or during handwashing**	Always	23 (10.6%)	60 (10.4%)	39 (9.2%)	0.0854
	Sometimes	23 (10.6%)	39 (6.7%)	19 (4.5%)	
	Never	12 (5.5%)	56 (9.7%)	29 (6.8%)	
	Not applicable *	160 (73.4%)	423 (73.2%)	339 (79.6%)	
**Using soap**	Always	206 (94.5%)	541 (93.6%)	399 (93.7%)	0.3960
	Sometimes	10 (4.6%)	34 (5.9%)	27 (6.3%)	
	Never	2 (0.9%)	3 (0.5%)	0 (0%)	
**Using warm water**	Always	140 (58.1%)	371 (58.2%)	253 (54.4%)	0.4848
	Sometimes	74 (30.7%)	195 (30.6%)	160 (34.4%)	
	Never	4 (1.7%)	12 (1.9%)	13 (2.8%)	
**Soaking hands before using soap**	Always	153 (70.2%)	408 (70.6%)	318 (74.6%)	0.1480
	Sometimes	34 (15.6%)	104 (18%)	74 (17.4%)	
	Never	31 (14.2%)	64 (11.1%)	34 (8.0%)	
	Not applicable *	0 (0%)	2 (0.3%)	0 (0%)	
**Careful making soap lather on whole hands**	Always	138 (63.3%)	334 (57.8%)	264 (62%)	0.3941
	Sometimes	74 (33.9%)	213 (36.9%)	143 (33.6%)	
	Never	6 (2.8%)	30 (5.2%)	19 (4.5%)	
	Not applicable *	0 (0%)	1 (0.2%)	0 (0%)	
**Turning the faucet off with hand**	Always	114 (52.3%)	301 (52.1%)	201 (47.2%)	0.1880
	Sometimes	55 (25.2%)	143 (24.7%)	134 (31.4%)	
	Never	49 (22.5%)	134 (23.2%)	91 (21.4%)	
**Drying hands with towel**	Always	180 (82.6%)	477 (82.5%)	332 (77.9%)	0.2588
	Sometimes	31 (14.2%)	86 (14.9%)	82 (19.2%)	
	Never	7 (3.2%)	15 (2.6%)	12 (2.8%)	

* not included to statistical analysis.

## Supplementary Materials

The following are available online at https://www.mdpi.com/2076-0817/9/12/1011/s1, Table S1. The declared handwashing procedure in the studied national sample of adolescents, in regions stratified by Gross Domestic Product (GDP) (comparison conducted while using chi^2^ test). Table S2. The declared circumstances of washing hands, on the basis of the Handwashing Habits Questionnaire, in the studied national sample of adolescents, in regions stratified by COVID-19 morbidity, Gross Domestic Product (GDP) and the size of the town. Table S3. The declared handwashing procedure in the studied national sample of adolescents, in regions stratified by COVID-19 morbidity, Gross Domestic Product (GDP) and the size of the town.

## References

[1] Ali I., Alharbi O.M. (2020). COVID-19: Disease, management, treatment, and social impact. Sci. Total Environ..

[2] Li H., Liu S.M., Yu X.H., Tang S.L., Tang C.K. (2020). Coronavirus disease 2019 (COVID-19): Current status and future perspective. Int. J. Antimicrob. Agents.

[3] World Health Organization (WHO) Statement on the second meeting of the International Health Regulations (2005) Emergency Committee Regarding the Outbreak of Novel Coronavirus (2019-nCoV). https://www.who.int/news-room/detail/30-01-2020-statement-on-the-second-meeting-of-the-international-health-regulations-(2005)-emergency-committee-regarding-the-outbreak-of-novel-coronavirus-(2019-ncov).

[4] World Health Organization (WHO) WHO Director-General’s Opening Remarks at the Media Briefing on COVID-19—11 March 2020. https://www.who.int/dg/speeches/detail/who-director-general-s-opening-remarks-at-the-media-briefing-on-covid-19---11-march-2020.

[5] European Centre for Disease Prevention and Control Transmission of COVID-19. https://www.ecdc.europa.eu/en/covid-19/latest-evidence/transmission.

[6] Buonanno G., Morawska L., Stabile L. (2020). Quantitative assessment of the risk of airborne transmission of SARS-CoV-2 infection: Prospective and retrospective applications. Environ. Int..

[7] Lotfi M., Hamblin M.R., Rezaei N. (2020). COVID-19: Transmission, prevention, and potential therapeutic opportunities. Clin. Chim. Acta.

[8] Chu D.K., Akl E.A., Duda S., Solo K., Yaacoub S., Schünemann H.J., on behalf of the COVID-19 Systematic Urgent Review Group Effort (SURGE) study authors (2020). Physical distancing, face masks, and eye protection to prevent person-to-person transmission of SARS-CoV-2 and COVID-19: A systematic review and meta-analysis. Lancet.

[9] Liang M., Gao L., Cheng C., Zhou Q., Uy J.P., Heiner K., Sun C. (2020). Efficacy of face mask in preventing respiratory virus transmission: A systematic review and meta-analysis. Travel Med. Infect. Dis..

[10] Morawska L., Tang J.W., Bahnfleth W., Bluyssen P.M., Boerstra A., Buonanno G., Cao J., Dancer S., Floto A., Franchimon F. (2020). How can airborne transmission of COVID-19 indoors be minimised?. Environ. Int..

[11] Buonanno G., Stabile L., Morawska L. (2020). Estimation of airborne viral emission: Quanta emission rate of SARS-CoV-2 for infection risk assessment. Environ. Int..

[12] Morawska L., Milton D.K. (2020). It Is Time to Address Airborne Transmission of Coronavirus Disease 2019 (COVID-19). Clin. Infect. Dis..

[13] Alzyood M., Jackson D., Aveyard H., Brooke J. (2020). COVID—19 reinforces the importance of hand washing. J. Clin. Nurs..

[14] Cherrie J.W., Loh M., Aitken R.J. (2020). Protecting healthcare workers from inhaled SARS-CoV-2 virus. Occup. Med..

[15] Spinazzè A., Cattaneo A., Cavallo D.M. (2020). COVID-19 outbreak in Italy: Protecting worker health and the response of the Italian Industrial Hygienists Association. Ann. Work Expo. Health.

[16] Vishnevetsky A., Levy M. (2020). Rethinking high-risk groups in COVID-19. Mult. Scler. Relat. Disord..

[17] Efuribe C., Barre-Hemingway M., Vaghefi E., Suleiman A.B. (2020). Coping with the COVID-19 crisis: A call for youth engagement and the inclusion of young people in matters that affect their lives. J. Adolesc. Health.

[18] Mantovani A., Rinaldi E., Zusi C., Beatrice G., Saccomani M.D., Dalbeni A. (2020). Coronavirus disease 2019 (COVID-19) in children and/or adolescents: A meta-analysis. Pediatr. Res..

[19] Kar S.K., Verma N., Saxena S.K. (2020). Coronavirus Infection Among Children and Adolescents. Coronavirus Dis. 2019 (COVID-19).

[20] Park Y.J., Choe Y.J., Park O., Park S.Y., Kim Y.M., Kim J., Kweon S., Woo Y., Gwack J., Kim S.S. (2020). Contact tracing during coronavirus disease outbreak, South Korea. Emerging Infect. Dis..

[21] World Health Organization (WHO) WHO Save Lives: Clean Your Hands in the Context of COVID-19. https://www.who.int/infection-prevention/campaigns/clean-hands/WHO_HH-Community-Campaign_finalv3.pdf?ua=1.

[22] Centers for Disease Control and Prevention (CDC) When and How to Wash Your Hands. https://www.cdc.gov/handwashing/when-how-handwashing.html.

[23] Chen X., Ran L., Liu Q., Hu Q., Du X., Tan X. (2020). Hand Hygiene, Mask-Wearing Behaviors and Its Associated Factors during the COVID-19 Epidemic: A Cross-Sectional Study among Primary School Students in Wuhan, China. Int. J. Environ. Res. Public Health.

[24] Ubheeram J., Biranjia-Hurdoyal S.D. (2017). Effectiveness of hand hygiene education among a random sample of women from the community. J. Prev. Med. Hyg..

[25] Wong J.S.W., Lee J.K.F. (2019). The common missed handwashing instances and areas after 15 years of hand-hygiene education. J. Environ. Public Health.

[26] Suen L.K., So Z.Y., Yeung S.K., Lo K.Y., Lam S.C. (2019). Epidemiological investigation on hand hygiene knowledge and behaviour: A cross-sectional study on gender disparity. BMC Public Health.

[27] Anderson J.L., Warren C.A., Perez E., Louis R.I., Phillips S., Wheeler J., Cole M., Misra R. (2008). Gender and ethnic differences in hand hygiene practices among college students. Am. J. Infect. Control..

[28] Van de Mortel T., Bourke R., McLoughlin J., Nonu M., Reis M. (2001). Gender influences handwashing rates in the critical care unit. Am. J. Infect. Control..

[29] Mariwah S., Hampshire K., Kasim A. (2012). The impact of gender and physical environment on the handwashing behaviour of university students in Ghana. Trop. Med. Int. Health.

[30] Dobe M., Mandal R.N., Jha A. (2013). Social determinants of good hand-washing practice (GHP) among adolescents in a rural Indian community. Fam. Community Health.

[31] Peltzer K., Pengpid S. (2014). Oral and hand hygiene behaviour and risk factors among in-school adolescents in four Southeast Asian countries. Int. J. Environ. Res. Public Health.

[32] Qorbani M., Kelishadi R., Djalalinia S., Motlagh M.E., Kasaeian A., Ardalan G., Shafiee G., Safari O., Heshmat R., Mahdavi S.B. (2016). Regional disparity in hygienic behaviors of Iranian children and adolescents: The CASPIAN-IV study. Med. J. Islam Repub. Iran..

[33] Taddese A.A., Dagnew B., Dagne H., Andualem Z. (2020). Mother’s Handwashing Practices and Health Outcomes of Under-Five Children in Northwest Ethiopia. Pediatric Health Med. Ther..

[34] To K.G., Lee J.K., Nam Y.S., Trinh O.T.H., Do D.V. (2016). Hand washing behavior and associated factors in Vietnam based on the Multiple Indicator Cluster Survey, 2010–2011. Glob. Health Action.

[35] Aiello A.E., Coulborn R.M., Perez V., Larson E.L. (2008). Effect of hand hygiene on infectious disease risk in the community setting: A meta-analysis. Am. J. Public Health.

[36] Moore L.D., Robbins G., Quinn J., Arbogast J.W. (2020). The Impact of COVID-19 Pandemic on Hand Hygiene Performance in Hospitals. Am. J. Infect. Control.

[37] Głąbska D., Skolmowska D., Guzek D. (2020). Population-Based Study of the Influence of the COVID-19 Pandemic on Hand Hygiene Behaviors—Polish Adolescents’ COVID-19 Experience (PLACE-19) Study. Sustainability.

[38] Guzek D., Skolmowska D., Głąbska D. (2020). Analysis of Gender-Dependent Personal Protective Behaviors in a National Sample: Polish Adolescents’ COVID-19 Experience (PLACE-19) Study. Int. J. Environ. Res. Public Health.

[39] Głąbska D., Skolmowska D., Guzek D. (2020). Population-Based Study of the Changes in the Food Choice Determinants of Secondary School Students: Polish Adolescents’ COVID-19 Experience (PLACE-19) Study. Nutrients.

[40] World Health Organization (WHO) Coronavirus Disease (COVID-19) Weekly Epidemiological Update and Weekly Operational Update. https://www.who.int/emergencies/diseases/novel-coronavirus-2019/situation-reports/?gclid=CjwKCAjwq_D7BRADEiwAVMDdHvneRLIPC03U3FJa-UNKxxjMd7TP28noIrDy_k9iqdH6_ns_ns6_ns_k9iqdH6_ns.

[41] Polish Ministry of Health https://www.gov.pl/web/koronawirus/wykaz-zarazen-koronawirusem-sars-cov-2.

[42] The Central Statistical Office in Poland (2019). https://bdl.stat.gov.pl/BDL/dane/podgrup/temat.

[43] Polish Ministry of National Education https://www.gov.pl/web/edukacja/zawieszenie-zajec-w-szkolach.

[44] Mbroh L.A. (2019). Assessing Knowledge, Attitude and Practices of Hand Hygiene among University Students. Master’s Thesis.

[45] Sultana M., Mahumud R.A., Sarker A.R., Hossain S.M. (2016). Hand hygiene knowledge and practice among university students: Evidence from Private Universities of Bangladesh. Risk Manag. Healthc. Policy.

[46] Ergin A., Bostanci M., Onal O., Bozkurt A.I., Ergin N. (2011). Evaluation of students’ social hand washing knowledge, practices, and skills in a university setting. Cent. Eur. J. Public Health.

[47] Tüzün H., Karakaya K., Deniz E.B. (2015). Turkey Handwashing Survey: Suggestion for taking the ecological model into better consideration. Environ. Health Prev. Med..

[48] Üner S., Sevencan F., Başaran E., Balcı C., Bilaloğlu B. (2009). To determine some knowledge and attitudes related to the social hand washing of individuals who apply to a primary health center. TAF Prev. Med. Bull..

[49] World Health Organization (WHO) Hand Hygiene: Why, How & When?. https://www.who.int/gpsc/5may/Hand_Hygiene_Why_How_and_When_Brochure.pdf.

[50] (2009). WHO Guidelines on Hand Hygiene in Health Care: First Global Patient Safety Challenge Clean Care Is Safer Care. https://www.ncbi.nlm.nih.gov/books/NBK144035/.

[51] Centers for Disease Control and Prevention (CDC) Handwashing: A Healthy Habit in the Kitchen. https://www.cdc.gov/handwashing/handwashing-kitchen.html.

[52] Centers for Disease Control and Prevention (CDC) Proper Hygiene When around Animals. https://www.cdc.gov/healthywater/hygiene/etiquette/around_animals.html.

[53] United Nations International Children’s Emergency Fund Everything You Need to Know about Washing Your Hands to Protect against Coronavirus (COVID-19). https://www.unicef.org/coronavirus/everything-you-need-know-about-washing-your-hands-protect-against-coronavirus-covid-19.

[54] Eurostat GDP per Inhabitant in PPS (% of the EU-27 Average). https://ec.europa.eu/eurostat/web/regions/statistics-illustrated.

[55] The Central Statistical Office in Poland https://stat.gov.pl/cps/rde/xbcr/gus/oz_miasta_w_liczbach_2009_notatka_infor.pdf.

[56] The Central Statistical Office in Poland (2019). https://stat.gov.pl/obszary-tematyczne/ludnosc/ludnosc/powierzchnia-i-ludnosc-w-przekroju-terytorialnym-w-2019-roku,7,16.html.

[57] Kadi N., Khelfaoui M. (2020). Population density, a factor in the spread of COVID-19 in Algeria: Statistic study. Bull. Natl. Res. Cent..

[58] Rocklöv J., Sjödin H. (2020). High population densities catalyse the spread of COVID-19. J. Travel Med..

[59] Apisarnthanarak A., Apisarnthanarak P., Siripraparat C., Saengaram P., Leeprechanon N., Weber D.J. (2020). Impact of Anxiety and Fear for COVID-19 toward Infection Control Practices Among Thai Healthcare Workers. Infect. Control Hosp. Epidemiol..

[60] Harper C.A., Satchell L.P., Fido D., Latzman R.D. (2020). Functional fear predicts public health compliance in the COVID-19 pandemic. Int. J. Ment. Health Addict..

[61] Israel S., Harpaz K., Radvogin E., Schwartz C., Gross I., Mazeh H., Cohen M.J., Benenson S. (2020). Dramatically improved hand hygiene performance rates at time of coronavirus pandemic. Clin. Microbiol. Infect..

[62] McIntosh K., Hirsch M.S., Bloom A. (2020). Coronavirus Disease 2019 (COVID-19), UpToDate. https://www.uptodate.com/contents/coronavirus-disease-2019-covid-19-epidemiology-virology-and-prevention.

[63] Goumenou M., Sarigiannis D., Tsatsakis A., Anesti O., Docea A.O., Petrakis D., Tsoukalas D., Kostoff R., Rakitskii V., Spandidos D.A. (2020). COVID-19 in Northern Italy: An integrative overview of factors possibly influencing the sharp increase of the outbreak. Mol. Med. Rep..

[64] Eurostat Population Structure and Ageing. https://ec.europa.eu/eurostat/statistics-explained/index.php/Population_structure_and_ageing#Median_age_is_highest_in_Italy.

[65] The Central Statistical Office in Poland (2019). https://stat.gov.pl/obszary-tematyczne/ludnosc/ludnosc/ludnosc-stan-i-struktura-ludnosci-oraz-ruch-naturalny-w-przekroju-terytorialnym-stan-w-dniu-31-12-2019,6,27.html.

[66] Suk J.E., Semenza J.C. (2011). Future infectious disease threats to Europe. Am. J. Public Health.

[67] Rabbi S.E., Dey N.C. (2013). Exploring the gap between hand washing knowledge and practices in Bangladesh: A cross-sectional comparative study. BMC Public Health.

[68] Luby S.P., Halder A.K. (2008). Associations among handwashing indicators, wealth, and symptoms of childhood respiratory illness in urban Bangladesh. Trop. Med. Int. Health.

[69] You H., Wu X., Guo X. (2020). Distribution of COVID-19 Morbidity Rate in Association with Social and Economic Factors in Wuhan, China: Implications for Urban Development. Int. J. Environ. Res. Public Health.

[70] Takagi H., Kuno T., Yokoyama Y., Ueyama H., Matsushiro T., Hari Y., Ando T. (2020). Meta-regression of COVID-19 prevalence/fatality on socioeconomic characteristics of data from top 50 US large cities. J. Med. Virol..

[71] Sarmadi M., Marufi N., Moghaddam V.K. (2020). Association of COVID-19 global distribution and environmental and demographic factors: An updated three-month study. Environ. Res..

[72] Mane A.B., Reddy N.S., Reddy P., Chetana K.V., Srijith S.N., Sriniwas T. (2016). Differences of Hand Hygiene and its Correlates among School going Children in Rural and Urban Area of Karnataka, India. Arch. Med..

[73] Khan S., Kumar V., Priya N., Yadav S.S. (2017). Handwashing practices among the caregivers of under five children in rural and urban areas of Moradabad, India: A community based study. Int. J. Med. Sci. Public Health.

[74] Ranscombe P. (2020). Rural areas at risk during COVID-19 pandemic. Lancet Infect. Dis..

[75] Neiderud C.J. (2015). How urbanization affects the epidemiology of emerging infectious diseases. Infect. Ecol. Epidemiol..

