# Supplementary material for: Hand Hygiene Behaviors in a Representative Sample of Polish Adolescents in Regions Stratified by COVID-19 Morbidity and by Confounding Variables (PLACE-19 Study): Is There Any Association?
Source: Pathogens. 2020 Dec 1;9(12):1011. doi: 10.3390/pathogens9121011 (PMC7759844; doi:10.3390/pathogens9121011)
Supplement: Supplementary file 1 [file pathogens-09-01011-s001.pdf]

pathogens

MDPI

Table S1. The declared handwashing procedure in the studied national sample of adolescents, in regions stratified by Gross Domestic Product (GDP) (comparison conducted while using chi^2 test).

Characteristics		Low GDP in region	High GDP in region	*p*
**Folding sleeves**	Always	488 (51.0%)	133 (50.2%)	0.9768
	Sometimes	194 (20.3%)	55 (20.8%)	
	Never	40 (4.2%)	11 (4.1%)	
	Not applicable*	235 (24.5%)	66 (24.9%)	
**Removing watch and bracelets**	Always	120 (12.5%)	40 (15.1%)	0.2716
	Sometimes	176 (18.4%)	40 (15.1%)	
	Never	86 (9.0%)	27 (10.2%)	
	Not applicable*	575 (60.1%)	158 (59.6%)	
**Removing rings before or during handwashing**	Always	96 (10.0%)	26 (9.8%)	0.5927
	Sometimes	65 (6.8%)	16 (6.0%)	
	Never	72 (7.5%)	25 (9.4%)	
	Not applicable*	724 (75.7%)	198 (74.8%)	
**Using soap**	Always	897 (93.7%)	249 (94.0%)	0.5624
	Sometimes	57 (6.0%)	14 (5.3%)	
	Never	3 (0.3%)	2 (0.7%)	
**Using warm water**	Always	595 (62.2%)	169 (63.8%)	0.8142
	Sometimes	340 (35.5%)	89 (33.6%)	
	Never	22 (2.3%)	7 (2.6%)	
**Soaking hands before using soap**	Always	695 (72.7%)	184 (69.4%)	0.5635
	Sometimes	164 (17.1%)	48 (18.1%)	
	Never	97 (10.1%)	32 (12.1%)	
	Not applicable*	1 (0.1%)	1 (0.4%)	
**Careful making soap lather on whole hands**	Always	574 (60.0%)	162 (61.1%)	0.8130
	Sometimes	341 (35.6%)	89 (33.6%)	
	Never	42 (4.4%)	13 (4.9%)	
	Not applicable*	0 (0.0%)	1 (0.4%)	
**Turning the faucet off with hand**	Always	490 (51.2%)	126 (47.6%)	0.2107
	Sometimes	263 (27.5%)	69 (26.0%)	
	Never	204 (21.3%	70 (26.4%)	
**Drying hands with towel**	Always	767 (80.2%)	222 (83.8%)	0.3033
	Sometimes	163 (17.0%)	36 (13.6%)	
	Never	27 (2.8%)	7 (2.6%)	

*not included to statistical analysis

Table S2. The declared circumstances of washing hands, on the basis of the Handwashing Habits Questionnaire, in the studied national sample of adolescents, in regions stratified by COVID-19 morbidity, Gross Domestic Product (GDP) and the size of the town.

	**Higher frequency of washing hands in regions of**		
	**COVID-19 morbidity**	**GDP**	**Rural/urban environment**
Before meals	Low morbidity	-	Rural
After meals	Low morbidity	-	Rural
Before preparing meals	-	-	-
After preparing meals	Low morbidity	-	-
Before using the restroom	Low morbidity	High GDP	Rural
After using the restroom	Low morbidity	-	-
Before going to bed	-	-	-
After waking up in the morning	-	-	Rural
After combing their hair	Low morbidity	-	Rural
After coming back home	-	-	-
After using public transportation	-	-	-
After money exchange	-	-	-
When hands are visibly soiled	-	-	-
After handshaking	Low morbidity	Low GDP	Rural
After touching animals	Low morbidity	-	Rural
After handling animal waste	-	-	Rural
After handling animal food	-	-	-
After contacting babies	Low morbidity	-	-
After changing diapers	-	-	-
After blowing nose	Low morbidity	-	Rural
After sneezing	-	-	Rural
After coughing	-	-	Rural
Before touching sick people	-	-	Rural
After touching sick people	Low morbidity	Low GDP	-
After touching garbage	-	-	-
After cleaning their home	Low morbidity	Low GDP	-
After washing dishes	-	-	-
After doing laundry	-	-	-

Table S3. The declared handwashing procedure in the studied national sample of adolescents, in regions stratified by COVID-19 morbidity, Gross Domestic Product (GDP) and the size of the town.

	**Higher frequency of washing hands in regions of**		
	**COVID-19 morbidity**	**GDP**	**Rural/urban environment**
Folding sleeves	-	-	Rural
Removing watch and bracelets	Low morbidity	-	Rural
Removing rings before or during handwashing	Low morbidity	-	-
Using soap	-	-	-
Using warm water	-	-	-
Soaking hands before using soap	-	-	-
Careful making soap lather on whole hands	-	-	-
Turning the faucet off with hand	-	-	-
Drying hands with towel	Low morbidity	-	-
